# Natural history study of type 1 diabetes risk in adults (T1DRA): protocol for screening and follow-up of UK adults

**DOI:** 10.1136/bmjopen-2026-116354

**Published:** 2026-09-29

**Authors:** Rachel J Aitken, Ilana Kelland, Stu Toms, Clare Megson, Sarah Stollery, Ben Gillard, Zahra Hashmi, Jack Whitfield, Lilianna Bakinowska, Olivia Pearce, Vito Lampasona, Claire L Williams, Sian L Grace, Georgina L Mortimer, Anna E Long, Kathleen M Gillespie

**Affiliations:** 1 Diabetes and Metabolism, Bristol Medical School, University of Bristol, Bristol, UK; 2 Bristol Dental School, University of Bristol, Bristol, UK; 3 San Raffaele Diabetes Research Institute, IRCCS Ospedale San Raffaele, Milan, Italy

**Keywords:** Diabetes & endocrinology, Mass Screening, GENETICS, Immunology

## Abstract

**Abstract:**

**Introduction:**

Type 1 diabetes can be predicted through the presence of multiple circulating islet autoantibodies in the blood. However, although more than half of type 1 diabetes cases are diagnosed in adults, natural history and prediction studies to date have focused on children. This protocol describes the first type 1 diabetes natural history study in adults, both general population and relatives for screening with longitudinal monitoring.

**Methods and analysis:**

The study launched on 14 November 2023 and the planned end date is 31 May 2027. The recruitment target is 20 000 adults. Participants are consented online and posted capillary blood kits for remote self-sampling. Serum, isolated from samples returned to the laboratory by mail, is tested for islet autoantibodies in a two-step process. Samples are initially screened for autoantibodies to truncated (t)glutamate decarboxylase 65, insulinoma-associated antigen-2 and zinc transporter 8 using a multiplex luciferase immunoprecipitation system (LIPS) screening assay. Positive samples are retested in individual LIPS assays. Autoantibodies to insulin are measured by radiobinding assay. Individuals positive for one or more islet autoantibody are recontacted and a remote confirmation sample is requested.

Once positivity is confirmed, positive participants are provided education about the symptoms of type 1 diabetes and invited to annual monitoring to allow risk of future type 1 diabetes to be accurately measured. The confirmation sample is also tested for autoantibody characteristics including affinity and positivity by a second platform to allow a tiered approach to future follow-up.

**Ethics and dissemination:**

The study protocol was submitted to Liverpool Central Research Ethics Committee and received a favourable opinion on 26 October 2023. Results are disseminated through conference presentations, peer-reviewed journals and will be shared openly.

**STRENGTHS AND LIMITATIONS OF THIS STUDY**

- Online consent and remote sample collection are efficient and cost effective.
- High-performance luciferase immunoprecipitation system tests allow screening of very low volume blood samples.
- Participants are offered annual remote monitoring for type 1 diabetes staging.
- Samples will be interrogated to stratify risk.
- While education about diabetes is provided, this study does not include psychological support for participants.

## Introduction

 Type 1 diabetes (T1D) results from a breakdown in immune regulation that leads to expansion of autoreactive B cells as well as CD4+ and CD8+ T cells targeting the insulin-producing beta cells of the islets of Langerhans.^1^ The humoral response results in circulating autoantibodies to islet antigens including insulin (IAA),^2^ glutamic acid decarboxylase 65 (GADA)^3^/ truncated(t)GADA (tGADA),^4^ islet antigen-2 (IA-2A),^5^ zinc transporter 8 (ZnT8A)^6^ and more recently, tetraspanin 7.^7^ In children, developing multiple (≥2) islet autoantibodies before the age of 5 years confers >80% risk of developing diabetes by age 20.^8^ A screening study in Germany reported that 0.3% of children in the general population have two or more islet autoantibodies, highly predictive of future autoimmune diabetes.^9^ There is increasing evidence that children identified with multiple islet autoantibodies benefit because they are not diagnosed as an emergency in diabetic ketoacidosis.^10^

Despite the heterogeneity of T1D, current consensus classifies the prodrome to T1D as having three stages,^11^ with stages 1 and 2 being presymptomatic. Stage 1 is defined by the presence of multiple islet autoantibodies in the blood, without dysglycaemia; stage 2 by the presence of multiple islet autoantibodies in the blood with dysglycaemia. Stage 3 represents the onset of symptomatic T1D. Guidelines have been published for monitoring the stages of presymptomatic T1D^12–14^ to inform clinical diabetes specialists. Approval of Teplizumab,^15^ in the USA and UK to delay onset of T1D in individuals with stage 2 diabetes has increased focus on identifying individuals suitable for treatment with this new immunotherapy and for clinical trials of new therapies.

Recent studies in UK Biobank of all diagnoses of diabetes using a T1D Genetic Risk Score (GRS)^16^ showed that T1D occurs at a consistent rate in each decade of life and incidence studies show that more than half of T1D is diagnosed in adulthood.^17^

Islet autoimmunity has been identified in adult relatives of individuals with T1D; for instance, the Bart’s Oxford study recruited first-degree and second-degree relatives of all ages^18^ and the TrialNet Pathway to Prevention study recruits relatives up to 45 years.^19 20^ However, the risks for developing clinical T1D in adults across the age spectrum remain unclear and studies in the general population are rare. Recently, the results of screening 1087 adults (relatives and general population) from the Autoimmunity Screening for Kids study were reported.^21^ In total, 3.86% of adults screened positive for at least one islet autoantibody and 0.55% screened positive for multiple islet autoantibodies. When a second screening method was applied, only 1.75% were positive for a single islet autoantibody by both detection methods. Discordance in outcomes from different islet autoantibody platforms has been demonstrated recently.^22^ The recently published monitoring guidelines advise the ‘rule of twos’ to identify individuals in stage 1.^12^ This involves confirmation of multiple (two or more) islet autoantibody status in a second sample preferably in a laboratory that meets Islet Autoantibody Standardization Program (IASP) standards, and ideally using two independent methods. The key question for stage 1 participants in monitoring is ‘when will symptoms appear’? We previously studied a group of individuals labelled as ‘Slow Progressors’ because they had been positive for multiple islet autoantibodies for >10 years without developing symptoms. The Slow or non-Progressive Autoimmunity to the Islet of Langerhans study^23^ showed that ‘Slow Progressors’ existed in all five cohorts examined but there were no clear defining factors to identify those progressing slowly to symptomatic T1D. If islet autoimmunity occurs early in life, as birth cohorts suggest and Slow Progressors represent <20% of birth cohorts, there are important questions to be addressed about the natural history of adult-onset autoimmune diabetes^24^; for instance, does seroconversion occur in adulthood? In addition, as indicated in a recent review, it is unclear whether the T1D stages, as currently defined, are ideal for adults.^25^ In our previous studies of progression rate, we showed that some ‘Slow Progressors’ who were previously multiple islet autoantibody positive lost islet autoantibodies over time,^26^ suggesting that risk associated with single islet autoantibody positivity may be higher in some adults than in children.

### Study objectives

Overall, there is a critical gap in evidence related to the pathogenesis and natural history of T1D developing in adulthood. This is even more so in the general population where no studies focused on adults have been carried out. There are, therefore, many questions to be addressed.

The main objective of this natural history study is to consent 20 000 adults without a diagnosis of T1D, to collect capillary blood samples remotely and screen for four islet autoantibodies (tGADA, IAA, IA-2A and ZnT8A). Participants positive for single and multiple islet autoantibodies will be offered annual follow-up and monitoring. This will allow the following research questions to be addressed:

- What is the frequency of single and multiple islet autoantibody positivity in general population adults?
- Is risk estimation in general population adults different to that described in general population children or relatives of individuals with T1D?
- Do those who progress slowly from detectable islet autoimmunity to clinical symptoms also experience delayed time to commencing insulin treatment with higher levels of residual c-peptide post diagnosis?
- Is there seroconversion in adulthood?
- What protects adults with islet autoimmunity from developing diabetes in childhood?
- Do islet autoantibody characteristics (level, affinity, persistence, positivity by a second assay platform)? provide an opportunity for risk stratification in adults with islet autoantibodies?

### Definition of outcomes

#### Primary outcome

Prevalence of stages 1, 2, 3 T1D in UK adults.

#### Secondary outcomes

Risk associated with single islet autoantibody positivity and strategies to discriminate risk.

Islet autoantibody prevalence by age, rates of seroconversion, progression rates and metabolic changes in adults.

## Methods and analysis

The study is initially cross-sectional with a preliminary screen of serum from participants with subsequent longitudinal follow-up of individuals with one (prestage 1) or multiple (stages 1 and 2) islet autoantibodies.

### Study population

#### Inclusion criteria

Adults aged between 18 and 70 years, living in the UK (England, Scotland, Wales and Northern Ireland), and not already identified with presymptomatic or a diagnosis of insulin-requiring T1D, are eligible to participate in this study. Individuals with gestational diabetes, T2D and latent autoimmune diabetes in adults are not strictly excluded but are managed through a separate pathway to individuals who are diabetes free at study entry.

#### Exclusion criteria

Adults identified as unsuitable to participate following guidance of their clinician for instance those on immunosuppressive or blood-thinning medications.

The 18–70 years age range was chosen in discussion with the Ethics Committee to provide a broad age range of adults without requesting capillary blood samples from individuals over age 70 years for whom it may be more difficult to return samples by post.

### Advertising, enrolment, blood sampling and islet autoantibody measurement

The study was launched on World Diabetes Day, 14 November 2023, supported by Diabetes UK through online advertising followed by ongoing social media recruitment. Online recruitment can result in study populations that are not representative of the local population with decreased representation among older people, individuals with limited digital access and certain socioeconomically disadvantaged populations. To ensure diversity among participants, workplace and community recruitment is also taking place through posters in public spaces. Participants consent online through a dedicated website (T1DRA) with access to study documents including Participant Information Sheets in multiple languages. Those signing up are asked details of how they heard about the study to help guide future recruitment. Clinical data including age, height, weight, number of relatives with T1D and details of other autoimmune diseases are collected. Individuals with a diagnosis of other forms of diabetes (gestational or type 2 diabetes) are not excluded and treatment details including whether on insulin treatment are collected.

When full consent is obtained, participants are sent a kit by post containing all requirements to take a capillary blood sample (including two Sarstedt safety lancets 1.5 mm blade, 1.6 mm penetration, and a Sarstedt Microvette serum gel capillary tube with clotting activator (SARSTEDT AG & Co, Nümbrecht, Germany). Written instructions are provided (and video guidelines are available online). Blood samples up to a maximum volume of 200 µL are requested. Samples are returned to the laboratory in appropriate prepaid, tracked, packaging where serum is separated by centrifugation and serum volume recorded.

The type 1 diabetes risk in adults (T1DRA) islet autoantibody testing strategy involves testing in a multistep process for four islet autoantibodies (tGADA, IA-2A, ZnT8A and IAA), including screening and confirmation stages (figure 1).

**Figure 1:**
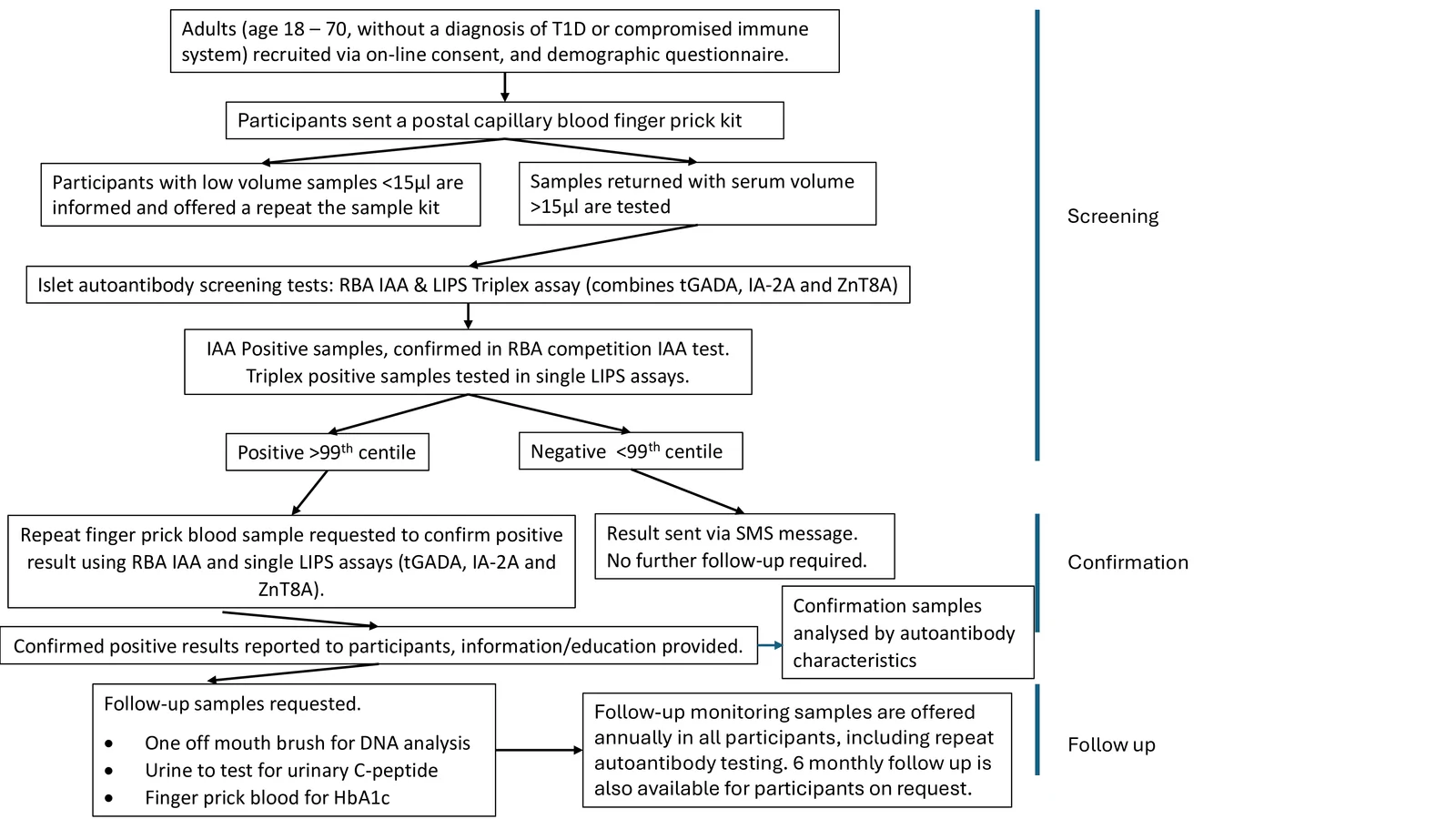
Flow diagram showing the participant journey and sample testing procedure in the T1DRA (Type 1 diabetes risk in adults) study. IAA, insulin autoantibodies; IA-2A, islet antigen-2 autoantibodies; LIPS, luciferase immunoprecipitation system; RBA, radiobinding assay; T1D, type 1 diabetes; ZnT8A, Zinc transporter-8 autoantibodies; HbA1c, glycated haenoglobin.

### tGADA/IA-2A/ZnT8A screening

Briefly, samples are initially screened for tGADA/IA-2A/ZnT8A in a triplex luciferase immunoprecipitation system assay that achieved an area under the receiver operator curve (area under the curve (AUC) receiver operating characteristic (ROC)) of 0.990 (95% CI 0.967 to 1.000, p<0.0001) in the IASP Workshop 2023. The adjusted sensitivity at 95% specificity was 98%. This low blood volume assay will indicate that samples are positive for one or more islet autoantibodies, but not the specificity of the autoantibodies. These samples are therefore retested in individual assays^27 28^ to determine antigen specificity and level.

Samples are then screened for IAA (on those not on insulin treatment) using a well-established radiobinding assay which includes an inherent screening step.^2^ Samples found positive in the screening stage undergo a competition stage (cIAA) to ensure specificity.

In all individual assays used to report results (tGADA, IA-2A, ZnT8A and cIAA), ‘positive’ was considered the 99th percentile of a healthy adult population.

### Initial communication with participants and confirmation testing

Individuals negative for tGADA/IA-2A/ZnT8A/IAA are notified by text and email message (“Dear T1DRA participant, you have tested negative for islet autoantibodies. This does not mean you will never get type 1 diabetes, but the chances are much lower. Thank you for helping us understand more about type 1 diabetes in adults. Visit our website for further updates. Kind regards, T1DRA study team*”*).

Individuals screening positive for one islet autoantibody are notified by email, explaining the result and the need for a confirmation capillary sample. Individuals screening positive for two or more islet autoantibodies are contacted by the T1DRA study nurse explaining the result and a confirmation capillary sample is requested.

Remote capillary collection kits are sent with instructions as previously described. Confirmation samples are returned to the laboratory, handled as described previously, except that signs of haemolysis are recorded using a Centers for Disease Control and Prevention (CDC) reference tool (https://www.cdc.gov/vector-borne-diseases/php/laboratories/reference-tool-for-hemolysis-status.html#cdc_generic_section_4-how-to-use-the-palette). Samples are stored at −20°C prior to analysis and tested in individual islet autoantibody assays.

When the islet autoantibody positive result has been confirmed in a separate sample, participants are again contacted by the study nurse who makes the participant aware of their confirmed islet autoantibody result and offers education about T1D. Participants are signposted to a growing number of pre-T1D clinics across the UK; to the UK Islet Autoantibody Registry (Homepage | UKIAb Registry) with access to psychological support and to clinical trials coordinated through the UK T1D Research Consortium (T1DUK – Type 1 Diabetes UK Immunotherapy Consortium).

### Monitoring islet autoantibody positive individuals

Once results are confirmed in two samples, all autoantibody positive participants (those with single and multiple islet autoantibodies) are offered a letter describing their results to share with their general practitioner/consultant if they so wish. All participants are invited to remote annual monitoring. If participants wish to, they can also request 6 monthly monitoring. A single mouth brush sample is taken for DNA (for Human Leucocyte Antigen (HLA) class I and II^29^ and T1D GRS-2^30^ analysis) and annual samples are taken for islet autoantibodies and metabolic monitoring through a capillary EDTA (SARSTEDT AG & Co) blood sample for HbA1c (glycated haemoglobin) levels. Urine samples are taken for urinary C peptide creatinine ratio.^31^

### Staging criteria

Staging criteria for T1D are recommended by the American Diabetes Association and British Society for Endocrinology and Diabetes.^12–14^ However, staging is based on risk estimates established in high-risk children. The aim of this study is to accurately establish risk in the general population adults, which are likely to be different. To communicate consistent advice to participants after metabolic monitoring by HbA1c, positive participants are staged as follows:

#### Stage 1

Confirmed positive for two or more islet autoantibodies with a HbA1c <42 mmol/mol.

#### Stage 2

Confirmed positive for two or more islet autoantibodies with a HbA1c 42–48 mmol/mol.

#### Stage 3

Confirmed positive for two or more islet autoantibodies with a HbA1c ≥48 mmol/mol.

This change to published monitoring guidelines^12–14^ is because of the increased levels of HbA1c observed in the general population UK adults.^32^ T1DRA is therefore using a higher threshold (≥42 mmol/mol (≥6%)), as suggested in a recent perspective focused on management of screen-detected islet autoantibody positivity in adults^33^ and aligning with glucose thresholds used to define non-diabetic range hyperglycaemia, advocated by WHO and used in many countries including the UK.

Individuals with a confirmed single islet autoantibody are also invited to monitoring. More adults are diagnosed with T1D with only a single islet autoantibody.^34^ It is therefore likely that adult screening will identify more single islet autoantibody positive individuals. Risk of future symptoms will be complicated as there are a few issues with communicating risk to single islet autoantibody positive individuals; some may be false positives, some may have transient single autoantibody positivity, some may have previously been positive for multiple islet autoantibodies and have lost positivity for some over time and therefore be at higher risk than those with persistent single islet autoantibody positivity.^26 35^ Discriminating risk among single islet autoantibody positive adults will be an important outcome from this study as the confirmation sample will be tested for islet autoantibody characteristics, including autoantibody level, epitopes, affinity and positivity by a second assay platform. If individuals are identified with low level, low affinity autoantibodies and are negative on a second platform, they will be made aware of their lower risk of T1D resulting in a tiered approach to follow-up.

This UK-wide study should be generalisable to other populations. Ethnicity data collected will allow comparison of positive and negative rates across diverse populations.

### Statistical analysis plan

Data will be analysed using well-established statistical programmes including SPSS and R as appropriate

- In this natural history study, we will investigate an older cohort than has previously been screened (TrialNet^19 20^ recruited first-degree relatives up to age 45 years only). The Fr1da study^9^ included general population screening of >90 000 children (age 2–5 years) in Bavaria, which showed that 3/1000 children had multiple islet autoantibodies. If autoantibodies are identified at half that rate across adulthood (age 18–70 years), and 20 000 adults were recruited, ~30 adults in stage 1 would be identified (and potentially more single islet autoantibody positives) for follow-up and risk estimation. In addition, relatives of individuals with T1D are not excluded and if recruited will increase the number of positive individuals.
- Prevalence and incidence estimation methods: the prevalence of islet autoimmunity will be presented as descriptive statistics. The prevalence of presymptomatic T1D will be determined as the percentage with two or more islet autoantibodies who were diabetes-free at study entry. This will be calculated separately for first-degree relatives, other relatives and for the general population.
- Prediction of progression rate to T1D in adults: Kaplan-Meier statistics will be used to evaluate the time-to-event distributions for time to progression to stage 3 T1D. Multivariable Cox regression models, time-dependent ROC curves and AUC measures will be used to evaluate the prognostic utility of measures of variables including age, sex, positive autoantibody number/combination/level/affinity/persistence/positivity by a second assay platform and HLA Class I and II/GRS. Where participants are lost to follow-up, two approaches will be used. Time to event analysis using (1) only complete data and (2) data censored at the end of follow-up. As survival analysis methods assume that participants censored and uncensored at a particular time with the same history have the same distribution of current and future variables, sensitivity analyses will be applied as described^36^.
- Longitudinal modelling: data collected from the same subjects over multiple time points will be analysed using mixed effects and additive models.
- An alpha value p<0.05 will be considered statistically significant for all analyses.
- Missing data handling: multiple imputation (MI) approaches will be utilised to cope with missingness in fitting multivariate time-series models. Variables in the T1DRA study that are likely to be missing are annual islet autoantibody values, annual HbA1c and notification of the date of diagnosis as well as missing data across all variables if participants have withdrawn from the study. These are crucial readouts and every effort will be made to obtain these data before analysis starts through direct communication with participants (where appropriate). If they cannot be obtained, MI will be used. We will consider whether too many data are missing to allow reliable imputation and whether this is random or non-random missingness. If there are sufficient data to proceed, best-worst and worst-best case sensitivity analyses will show the full theoretical range of uncertainty and conclusions will be related to this range of uncertainty.^37^

### Patient and public involvement

This study will identify general population adults with risk markers for T1D. There were no such individuals available to establish a Patient and Public Involvement and Engagement (PPIE) Group prior to the submission of the grant application. Therefore, the protocol was discussed with adult relatives of individuals with T1D who are known to have risk markers through another study.^18^ Moving forward, individuals who consent to participate in the study (adults with and without risk markers) will be invited to join a T1DRA study PPIE Group to inform future strategies. The group will meet quarterly and review all updates to study documentation and new collaborations linked to T1DRA. Feedback from the PPIE Group will be integrated into all future approaches.

### Risk and benefit evaluation

This is particularly important in light of results from a clinical trial published in 2018.^12^ The anti-CD3 drug, Teplizumab, was shown to delay the onset of clinical symptoms of diabetes for an average of 2 years in individuals in stage 2 T1D. As a result of this trial (with ongoing follow-up of participants), in November 2022, Teplizumab was licenced for use in the USA to delay onset of T1D in stage 2 individuals. More recently, the drug has been licenced by the Medicines and Healthcare products Regulatory Agency (MHRA) in the UK and in 2026, National Institute for Health and Care Excellence (NICE) approved National Health Service (NHS) use of Teplizumab to delay onset of T1D symptoms in individuals with stage 2 over the age of 8 years. Therefore, there is an international focus on T1D screening (Fr1DA^9^/EDENT1FI)).^38^ In the UK, the EarLy Surveillance for Autoimmune diabetes (ELSA) study^39^ is recruiting children aged 2–17 years from the general population and the UK Islet Autoantibody Registry is open. The T1DRA study will ensure that adults are also considered for screening, annual monitoring and opportunities to participate in clinical trials.

### Ethics and dissemination

National Research Ethics Committee approval was obtained on 26 October 2023 from Liverpool REC (Integrated Research Application System reference: 333462). REC reference: 23/NW/0305. The study outcomes will be disseminated through presentations at international conferences and publication in high-impact journals.

## References

[1] Willcox A, Richardson SJ, Bone AJ (2009). Analysis of islet inflammation in human type 1 diabetes. Clin Exp Immunol.

[2] Williams AJ, Bingley PJ, Bonifacio E (1997). A novel micro-assay for insulin autoantibodies. J Autoimmun.

[3] Baekkeskov S, Aanstoot HJ, Christgau S (1990). Identification of the 64K autoantigen in insulin-dependent diabetes as the GABA-synthesizing enzyme glutamic acid decarboxylase. Nature.

[4] Williams AJK, Lampasona V, Wyatt R (2015). Reactivity to N-Terminally Truncated GAD65(96-585) Identifies GAD Autoantibodies That Are More Closely Associated With Diabetes Progression in Relatives of Patients With Type 1 Diabetes. Diabetes.

[5] Payton MA, Hawkes CJ, Christie MR (1995). Relationship of the 37,000- and 40,000-M(r) tryptic fragments of islet antigens in insulin-dependent diabetes to the protein tyrosine phosphatase-like molecule IA-2 (ICA512). J Clin Invest.

[6] Wenzlau JM, Juhl K, Yu L (2007). The cation efflux transporter ZnT8 (Slc30A8) is a major autoantigen in human type 1 diabetes. Proc Natl Acad Sci U S A.

[7] McLaughlin KA, Richardson CC, Ravishankar A (2016). Identification of Tetraspanin-7 as a Target of Autoantibodies in Type 1 Diabetes. Diabetes.

[8] Ziegler AG, Rewers M, Simell O (2013). Seroconversion to multiple islet autoantibodies and risk of progression to diabetes in children. JAMA.

[9] Ziegler A-G, Kick K, Bonifacio E (2020). Yield of a Public Health Screening of Children for Islet Autoantibodies in Bavaria, Germany. JAMA.

[10] Rewers M (2025). Health economic considerations of screening for early type 1 diabetes. Diabetes Obes Metab.

[11] Insel RA, Dunne JL, Atkinson MA (2015). Staging presymptomatic type 1 diabetes: a scientific statement of JDRF, the Endocrine Society, and the American Diabetes Association. Diabetes Care.

[12] Phillip M, Achenbach P, Addala A (2024). Consensus Guidance for Monitoring Individuals With Islet Autoantibody-Positive Pre-Stage 3 Type 1 Diabetes. Diabetes Care.

[13] Leichter SB, Felton JL, Geno Rasmussen C (2025). Establishing Screening Programs for Presymptomatic Type 1 Diabetes: Practical Guidance for Diabetes Care Providers. J Clin Endocrinol Metab.

[14] Besser REJ, Campbell F, Damazer K (2025). UK best practice recommendations for children and young people <18 years with pre-stage 3 type 1 diabetes, on behalf of the British Society for Paediatric Endocrinology and Diabetes (BSPED). Diabet Med.

[15] Herold KC, Bundy BN, Long SA (2019). Type 1 Diabetes TrialNet Study Group. N Engl J Med.

[16] Thomas NJ, Jones SE, Weedon MN (2018). Frequency and phenotype of type 1 diabetes in the first six decades of life: a cross-sectional, genetically stratified survival analysis from UK Biobank. Lancet Diabetes Endocrinol.

[17] Rogers MAM, Kim C, Banerjee T (2017). Fluctuations in the incidence of type 1 diabetes in the United States from 2001 to 2015: a longitudinal study. BMC Med.

[18] Gillespie KM, Fareed R, Mortimer GL (2021). Four decades of the Bart’s Oxford study: Improved tests to predict type 1 diabetes. Diabet Med.

[19] Liu Y, Rafkin LE, Matheson D (2017). Use of self-collected capillary blood samples for islet autoantibody screening in relatives: a feasibility and acceptability study. Diabet Med.

[20] So M, O’Rourke C, Bahnson HT (2020). Autoantibody Reversion: Changing Risk Categories in Multiple-Autoantibody-Positive Individuals. Diabetes Care.

[21] Pauley ME, Simmons KM, Dong F (2025). Prevalence of Islet Autoantibodies in Adults Without Diabetes. J Endocr Soc.

[22] Krischer JP, Muller S, You L (2025). Comparative Analysis of the Sensitivity, Specificity, Concordance, and 5-Year Predictive Power of Diabetes-Related Autoantibody Assays. Diabetes.

[23] Long AE, Wilson IV, Becker DJ (2018). Characteristics of slow progression to diabetes in multiple islet autoantibody-positive individuals from five longitudinal cohorts: the SNAIL study. Diabetologia.

[24] Gillespie KM, Long AE (2019). What Have Slow Progressors Taught Us About T1D—Mind the Gap!. *Curr Diab Rep*.

[25] Evans-Molina C, Oram RA (2025). Type 1 diabetes presenting in adults: Trends, diagnostic challenges and unique features. Diabetes Obes Metab.

[26] Hanna SJ, Powell WE, Long AE (2020). Slow progressors to type 1 diabetes lose islet autoantibodies over time, have few islet antigen-specific CD8^+^ T cells and exhibit a distinct CD95^hi^ B cell phenotype. Diabetologia.

[27] Wyatt RC, Grace SL, Brigatti C (2024). Improved Specificity of Glutamate Decarboxylase 65 Autoantibody Measurement Using Luciferase-Based Immunoprecipitation System Assays. Diabetes.

[28] Williams CL, Marzinotto I, Brigatti C (2024). A novel, high-performance, low-volume, rapid luciferase immunoprecipitation system (LIPS) assay to detect autoantibodies to zinc transporter 8. Clin Exp Immunol.

[29] Lambert AP, Gillespie KM, Thomson G (2004). Absolute risk of childhood-onset type 1 diabetes defined by human leukocyte antigen class II genotype: a population-based study in the United Kingdom. J Clin Endocrinol Metab.

[30] Redondo MJ, Oram RA, Steck AK (2017). Genetic Risk Scores for Type 1 Diabetes Prediction and Diagnosis. Curr Diab Rep.

[31] Williams GM, Long AE, Wilson IV (2016). Beta cell function and ongoing autoimmunity in long-standing, childhood onset type 1 diabetes. Diabetologia.

[32] Rodgers LR, Hill AV, Dennis JM (2021). Choice of HbA1c threshold for identifying individuals at high risk of type 2 diabetes and implications for diabetes prevention programmes: a cohort study. BMC Med.

[33] Thomas N, Tatovic D, Jones A (2025). Managing adults with screen-detected islet autoantibody positivity: a pragmatic framework. Lancet Diabetes Endocrinol.

[34] Bravis V, Kaur A, Walkey HC (2018). Relationship between islet autoantibody status and the clinical characteristics of children and adults with incident type 1 diabetes in a UK cohort. ADDRESS-2 Management Committee, Patient Advocate Group and Investigators. BMJ Open.

[35] Achenbach P, Warncke K, Reiter J (2004). Stratification of type 1 diabetes risk on the basis of islet autoantibody characteristics. Diabetes.

[36] Reps JM, Rijnbeek P, Cuthbert A (2021). An empirical analysis of dealing with patients who are lost to follow-up when developing prognostic models using a cohort design. BMC Med Inform Decis Mak.

[37] Jakobsen JC, Gluud C, Wetterslev J (2017). When and how should multiple imputation be used for handling missing data in randomised clinical trials – a practical guide with flowcharts. *BMC Med Res Methodol*.

[38] Hoffmann L, Kohls M, Arnolds S (2025). EDENT1FI Master Protocol for screening of presymptomatic early-stage type 1 diabetes in children and adolescents. BMJ Open.

[39] Quinn LM, Dias RP, Greenfield SM (2025). Protocol for a feasibility and acceptability study for UK general population paediatric type 1 diabetes screening-the EarLy Surveillance for Autoimmune diabetes (ELSA) study. Diabet Med.

