# Supplementary material for: Natural history study of type 1 diabetes risk in adults (T1DRA): protocol for screening and follow-up of UK adults
Source: BMJ Open. 2026 Sep 29;16(9):e116354. doi: 10.1136/bmjopen-2026-116354 (PMC13630054; doi:10.1136/bmjopen-2026-116354)
Supplement: Supplementary file 1 [file bmjopen-2026-116354.reviewer_comments.pdf]

PEER REVIEW HISTORY

BMJ Open publishes all reviews undertaken for accepted manuscripts. Reviewers are asked to complete a checklist review form and are provided with free text boxes to elaborate on their assessment. These free text comments are reproduced below.

ARTICLE DETAILS

Title (Provisional)

Protocol for a natural history study of Type 1 Diabetes Risk in Adults (T1DRA): Screening and follow up of UK adults

Authors

Aitken, Rachel J; Kelland, Ilana; Toms, Stu; Megson, Clare; Stollery, Sarah; Gillard, Ben; Hashmi, Zahra; Whitfield, Jack; Bakinowska, Lilianna; Pearce, Olivia; Lampasona, Vito; Williams, Claire L; Grace, Sian L; Mortimer, Georgina L; Long, Anna E.; Gillespie, Kathleen

---

VERSION 1 - REVIEW

**Reviewer**	**1**
**Name**	**Bhowmik, Bishwajit**
**Affiliation**	**Diabetic Association of Bngladesh, Centre for Global Health Research**
**Date**	**23-Mar-2026**
**COI**	**None**

Reviewer Report to Authors

Manuscript ID: bmjopen-2026-116354

Title: Protocol for a natural history study of Type 1 Diabetes Risk in Adults (T1DRA)

General Comments

Thank you for the opportunity to review this important and well-conceived study protocol. This manuscript addresses a significant gap in the literature—namely, the natural history and prediction of type 1 diabetes (T1D) in adults, a population that has been historically underrepresented despite accounting for a substantial proportion of incident cases.

The study is novel, timely, and methodologically innovative, particularly due to its large-scale recruitment, remote screening approach, and integration of immunological, genetic, and metabolic markers. The protocol is generally well written and aligned with the aims of BMJ Open.

However, several areas require clarification and strengthening to ensure methodological transparency and reproducibility.

Major Comments

1. Statistical Analysis Plan (Critical)

The protocol currently lacks a sufficiently detailed statistical analysis plan.

• There is no clear description of:

o Sample size justification

o Planned statistical models

o Handling of missing data

o Longitudinal analysis strategy

Recommendation:

Please include a dedicated “Statistical Analysis” section detailing:

• Prevalence and incidence estimation methods

• Time-to-event analysis (e.g., Cox proportional hazards models)

• Longitudinal modeling (e.g., mixed-effects models)

• Missing data handling (e.g., multiple imputation)

• Sensitivity analyses

2. Definition of Outcomes

The manuscript does not clearly define primary and secondary outcomes.

Recommendation:

Explicitly specify:

• Primary outcome: Incidence of clinically diagnosed T1D

• Secondary outcomes: Autoantibody prevalence, progression rates, seroconversion, metabolic changes, etc.

3. Potential Selection Bias

Recruitment via online platforms and Diabetes UK advertising may introduce selection bias.

Concerns:

• Overrepresentation of:

o Health-conscious individuals

o Higher socioeconomic groups

Recommendation:

• Discuss representativeness explicitly

• Describe how recruitment source data will be used to assess bias

4. Psychological Impact and Ethical Considerations

The protocol acknowledges that psychological support is not included.

Concern:

Disclosure of risk status may cause anxiety or distress.

Recommendation:

• Provide a mitigation strategy, such as:

o Counseling pathways

o Referral mechanisms

o Clear communication protocols

5. Justification of Modified Staging Criteria

The study applies staging criteria based on HbA1c that differ from standard ADA/ISPAD definitions.

Recommendation:

• Provide stronger justification

• Clarify how results will be interpreted relative to established staging systems

Minor Comments

6. Inclusion of Participants with Type 2 Diabetes

Including participants with other diabetes types may introduce heterogeneity.

Suggestion:

Clarify:

• Diagnostic differentiation (e.g., LADA vs T2D)

• Planned subgroup analyses

7. Follow-up Schedule

The follow-up frequency is described as both annual and six-monthly.

Recommendation:

Clarify whether follow-up is:

• Fixed

• Risk-stratified

8. Data Management and Quality Assurance

While GDPR compliance is mentioned, operational details are limited.

Suggestion:

Add:

• Data quality control procedures

• Monitoring processes

• Data validation strategies

9. Flow Diagram

The protocol references a flow diagram but lacks detailed description.

Recommendation:

Ensure the diagram clearly illustrates:

• Screening

• Confirmation

• Follow-up pathways

10. Patient and Public Involvement (PPI)

PPI is limited at the protocol stage.

Suggestion:

Expand on:

• Future engagement plans

• Integration of participant feedback

Strengths of the Study

• First large-scale adult population-based T1D screening study

• Innovative remote sampling and digital recruitment model

• Robust multi-step autoantibody testing strategy

• High potential for clinical and policy impact

• Alignment with emerging therapies (e.g., teplizumab)

Conclusion

This is a high-quality and impactful study protocol that addresses a major gap in diabetes research. With revisions to improve clarity, methodological transparency, and ethical considerations, the manuscript will make a valuable contribution to the field.

Recommendation to Authors:

Major Revision

VERSION 1 - AUTHOR RESPONSE

Reviewer: 1

Several areas require clarification and strengthening to ensure methodological transparency and reproducibility.
Response: we agree and are grateful to the Reviewer for highlighting the specific areas where improvement is needed. We have endeavoured to do this as fully as possible.

1. Statistical Analysis Plan (Critical)
Recommendation:
Please include a dedicated “Statistical Analysis” section detailing:
• Prevalence and incidence estimation methods
• Time-to-event analysis (e.g., Cox proportional hazards models)
• Longitudinal modeling (e.g., mixed-effects models)
• Missing data handling (e.g., multiple imputation)
• Sensitivity analyses

Response: Thank you. There was a brief data analysis plan in the original manuscript. This has been retitled as the Statistical analysis plan now on Page 11 and expanded as suggested by the Reviewer.

2. Definition of Outcomes
The manuscript does not clearly define primary and secondary outcomes.
Recommendation:
Explicitly specify:
• Primary outcome: Incidence of clinically diagnosed T1D
• Secondary outcomes: Autoantibody prevalence, progression rates, seroconversion, metabolic changes, etc.

Response: Thank you. A definition of outcomes section has been added to Page 7

3. Potential Selection Bias
Recruitment via online platforms and Diabetes UK advertising may introduce selection bias.
Concerns:
• Overrepresentation of:
o Health-conscious individuals
o Higher socioeconomic groups
Recommendation:
• Discuss representativeness explicitly
• Describe how recruitment source data will be used to assess bias
Response: The Reviewer is absolutely correct recruiting through social media and charities related to diabetes results in more relatives of people with diabetes and more White females. The study has therefore also worked on targeted social media posts for different ethnicities; workplace and community recruitment – this has been added to the manuscript Page 8.

4. Psychological Impact and Ethical Considerations
The protocol acknowledges that psychological support is not included.

Concern:
Disclosure of risk status may cause anxiety or distress.
Recommendation:
• Provide a mitigation strategy, such as:
o Counselling pathways
o Referral mechanisms
o Clear communication protocols

Response: This is an important point and we know different people interpret risk differently. Although psychological support is not a funded part of this project, the participants are contacted by the study nurse who has decades of experience in delivering the type 1 diabetes risk message. She is also able to signpost participants to an increasing number of consultant-led adult pre-type 1 diabetes clinics across the UK. In addition participants are made aware of the UK Islet autoantibody registry with access to psychological support. This information has been added to Page 10.

5. Justification of Modified Staging Criteria
The study applies staging criteria based on HbA1c that differ from standard ADA/ISPAD definitions.
Recommendation:
• Provide stronger justification
• Clarify how results will be interpreted relative to established staging systems

Response: Apologies for any confusion, the staging in the submitted manuscript follows the published consensus monitoring guidelines Consensus Guidance for Monitoring Individuals With Islet Autoantibody-Positive Pre-Stage 3 Type 1 Diabetes - PubMed

Stage 1: HbA1c <39 mmol/mol (<5.7%)

Stage 2: HbA1c 39–47mmol/mol (5.7–6.4%)

Stage 3: HbA1c ≥48mmol/mol (≥6.5%)

However in adults, we are using the framework published by our collaborators Managing adults with screen-detected islet autoantibody positivity: a pragmatic framework - PubMed

This is because of the increased levels of HbA1c observed in general population adults as described here – 40% of general population adults had HbA1c >39 mmol/mol (<5.7%) Choice of HbA1c threshold for identifying individuals at high risk of type 2 diabetes and implications for diabetes prevention programmes: a cohort study | BMC Medicine | Springer Nature Link

We are therefore using a higher threshold (≥42 mmol/mol [≥6%]), aligning with glucose thresholds used to define non-diabetic range hyperglycaemia advocated by WHO and used in many countries, including the UK Use of glycated haemoglobin (HbA1c) in diagnosis of diabetes mellitus

This information has been updated in the manuscript page 11.

Minor Comments

6. Inclusion of Participants with Type 2 Diabetes
Including participants with other diabetes types may introduce heterogeneity.

Suggestion:
Clarify:
• Diagnostic differentiation (e.g., LADA vs T2D);
• Planned subgroup analyses

Response: We agree. The reason the only exclusion was type 1 diabetes was to allow preliminary data to be collected on islet autoantibody positivity in those with other forms of diabetes. Page 7 has been updated to explain the inclusion and exclusion criteria.

7. Follow-up Schedule
The follow-up frequency is described as both annual and six-monthly.
Recommendation:
Clarify whether follow-up is:
• Fixed
• Risk-stratified
Response: When we initiated the study follow up was annual for all individuals with single or multiple islet autoantibodies were offered annual follow up. Some individuals with islet autoantibodies requested the opportunity for 6 months follow up. Our ethical approval was updated to allow this. All participants are now made aware of 6 monthly follow up but only a subset avail of it. The manuscript has been updated to clarify this. In addition, in the confirmation study we carry out additional testing as described on the top of page 12.

8. Data Management and Quality Assurance
While GDPR compliance is mentioned, operational details are limited.
Suggestion:
Add:
• Data quality control procedures
• Monitoring processes
• Data validation strategies

Response:

Thank you. We have updated the Data management and quality assurance section on Page 15 as requested.

9. Flow Diagram
The protocol references a flow diagram but lacks detailed description.
Recommendation:
Ensure the diagram clearly illustrates:

• Screening
• Confirmation
• Follow-up pathways

Response: Figure 1 has been updated to highlight the screening,

10. Patient and Public Involvement (PPI)
PPI is limited at the protocol stage.
Suggestion:
Expand on:
• Future engagement plans
• Integration of participant feedback
Response: We have updated the PPI section on page 13 to include more detail on how PPIE is integrated into the T1DRA study.

Strengths of the Study
• First large-scale adult population-based T1D screening study
• Innovative remote sampling and digital recruitment model
• Robust multi-step autoantibody testing strategy
• High potential for clinical and policy impact
• Alignment with emerging therapies (e.g., teplizumab)
Conclusion
This is a high-quality and impactful study protocol that addresses a major gap in diabetes research. With revisions to improve clarity, methodological transparency, and ethical considerations, the manuscript will make a valuable contribution to the field.

Response: thank you; your suggestions have been much appreciated.

VERSION 2 - REVIEW

**Reviewer**	**1**
**Name**	**Bhowmik, Bishwajit**
**Affiliation**	**Diabetic Association of Bngladesh, Centre for Global Health Research**
**Date**	**11-Jul-2026**
**COI**	

Thank you for the opportunity to review the revised version of your manuscript entitled "Protocol for a natural history study of Type 1 Diabetes Risk in Adults (T1DRA): Screening and follow up of UK adults."

I appreciate the considerable effort you have invested in revising the manuscript and addressing the previous reviewer comments. Your responses are comprehensive, and the revised manuscript has improved substantially in terms of methodological clarity, transparency, and overall presentation.

In particular, I commend the authors for:

• expanding the Statistical Analysis Plan to include prevalence estimation, survival analysis, longitudinal modelling, handling of missing data, and sensitivity analyses;

• clearly defining the primary and secondary study outcomes;

• strengthening the ethical framework by describing participant counselling, referral pathways, and access to psychological support through specialist services;

• improving the description of recruitment strategies and acknowledging potential selection bias;

• providing a clearer rationale for the modified staging criteria used for adult participants;

• expanding the Patient and Public Involvement section; and

• improving the participant flow and follow-up procedures.

These revisions have significantly strengthened the manuscript and increase confidence in the reproducibility and scientific value of the proposed study.

I have only a few minor suggestions that may further improve the manuscript before publication:

1. Please provide a brief statistical justification for the planned recruitment target of 20,000 participants, including the expected prevalence of islet autoantibody positivity and anticipated number of outcome events.

2. Although the discussion of recruitment bias has improved, it would be useful to acknowledge that online recruitment may continue to underrepresent older adults, individuals with limited digital access, and certain socioeconomically disadvantaged populations.

3. Please clarify the assumptions underlying the planned multiple imputation approach (e.g., missing at random) and briefly indicate the variables that will be included in the imputation model.

4. A brief statement describing how participants lost to follow-up will be handled analytically (for example, censoring within survival analyses and planned sensitivity analyses) would further strengthen the statistical methods.

5. Please specify the planned statistical significance level (e.g., two-sided $P < 0.05$) and confidence intervals used throughout the analyses.

6. Consider adding one or two sentences discussing the generalisability of the findings beyond the UK population.

7. If feasible, Figure 1 could be further enhanced by indicating the anticipated number of participants at each major stage of screening and follow-up.

8. Finally, I recommend a careful editorial proofread to correct a few minor typographical and formatting inconsistencies (for example, spacing around units, punctuation, and uniform terminology).

Overall, I believe this is a well-designed, innovative, and highly important study protocol that addresses a major knowledge gap regarding adult-onset Type 1 diabetes. The revised manuscript is scientifically sound and is likely to have substantial impact on future screening strategies and clinical research. I congratulate the authors on an excellent revision and look forward to seeing this work published.

I recommend acceptance after minor revision.

VERSION 2 - AUTHOR RESPONSE

Thank you again to the Reviewer for very helpful comments. The responses and changes to the manuscript are provided below.

1. Please provide a brief statistical justification for the planned recruitment target of 20,000 participants, including the expected prevalence of islet autoantibody positivity and anticipated number of outcome events.
Response: It was difficult to decide how many general adult participants to recruit for this natural history study given that we are investigating an older cohort than has previously been screened (TrialNet recruited first degree relative up to age 45 years only). The Fr1da study included general population screening of >90,000 children (age 3-5) in Bavaria showed that 3/1000 children had multiple islet autoantibodies. If autoantibodies were identified at half that rate, screening 20,000 adults would allow us to identify 30 adults in Stage 1 for follow up and potentially more adults with single islet autoantibodies. The decision to screen 20,000 adults was a combination of pragmatism in terms of numbers of people screened and identifying sufficient individuals for follow-up to type 1 diabetes to allow robust risk calculations.
Page 13*: In this natural history study, we will investigate an older cohort than has previously been screened (TrialNet [12] recruited first degree relative up to age 45 years only). The Fr1da study [9] included general population screening of >90,000 children (age 2-5 years) in Bavaria showed that 3/1000 children had multiple islet autoantibodies. If autoantibodies are identified at half that rate across adulthood (age 18-70 years), and 20,000 adults were recruited, ~30 adults in Stage 1 would be identified (and potentially more single islet autoantibody positives) for follow up. In addition relatives of individuals with type 1 diabetes are not excluded and if recruited will increase the number of positive individuals.*

2. Although the discussion of recruitment bias has improved, it would be useful to acknowledge that online recruitment may continue to underrepresent older adults, individuals with limited digital access, and certain socioeconomically disadvantaged populations.
Response: we acknowledge that online recruitment has limitations and have added the following section to page 8: *Online recruitment can result in study populations that are not representative of the local population with decreased representation among older people,*

individuals with limited digital access, and certain socioeconomically disadvantaged populations.

3. Please clarify the assumptions underlying the planned multiple imputation approach (e.g., missing at random) and briefly indicate the variables that will be included in the imputation model.
Response: we plan to use the approaches outlined by Jakobsen and colleagues (When and how should multiple imputation be used for handling missing data in randomised clinical trials – a practical guide with flowcharts | BMC Medical Research Methodology | Springer Nature Link
Variables in the T1DRA study that are likely to be missing are longitudinal islet autoantibody values, annual HbA1c and notification of the date of diagnosis. These are crucial readouts and every effort will be made to obtain these data before analysis starts through direct communication with participants (where appropriate). If they cannot be obtained, multiple imputation will be used where possible. Initially we will consider whether too many data are missing to allow reliable imputation whether this is random or non-random missingness. If there are sufficient data to proceed, best- and worst-best case sensitivity analyses will be applied to show the range of uncertainty and our conclusions will be related to this range of uncertainty.

4. A brief statement describing how participants lost to follow-up will be handled analytically (for example, censoring within survival analyses and planned sensitivity analyses) would further strengthen the statistical methods.
Response: T1DRA survival analysis will be based on Stage 3 T1D as the outcome. Where participants are lost to follow up, two approaches will be used. Time to event analysis using 1. only complete data and 2. data censored at end of follow up. As survival analysis methods assume that participants censored and uncensored at a particular time with the same history have the same distribution of current and future variables, sensitivity analyses will be applied as described. (An empirical analysis of dealing with patients who are lost to follow-up when developing prognostic models using a cohort design | BMC Medical Informatics and Decision Making | Springer Nature Link). This has been added to Page 13.

5. Please specify the planned statistical significance
Response: $P < 0.05$ will be considered as statistical significance although the actual p values will be provided to allow independent interpretation. This has been added to Page 13.

6. Consider adding one or two sentences discussing the generalisability of the findings beyond the UK population.
Response: The following sentences have been added to Page 12
This UK wide study should be generalisable to other populations. Ethnicity data collected will allow comparison of positive and negative rates across diverse populations.

7. If feasible, Figure 1 could be further enhanced by indicating the anticipated number of participants at each major stage of screening and follow-up.
Response: We understand the request, but we would prefer not to do this because we have gathered sufficient data since recruitment began to be aware that our predictions would not have been accurate particularly for single islet autoantibody positivity.

8. Finally, I recommend a careful editorial proofread to correct a few minor typographical and formatting inconsistencies (for example, spacing around units, punctuation, and uniform terminology).
Response: Thank you – we have proofread the draft.

Overall, I believe this is a well-designed, innovative, and highly important study protocol that addresses a major knowledge gap regarding adult-onset Type 1 diabetes. The revised manuscript is scientifically sound and is likely to have substantial impact on future screening strategies and clinical research. I congratulate the authors on an excellent revision and look forward to seeing this work published.

Response: Thank you
